# Opioid Sparing Multimodal Analgesia for Transoral Robotic Surgery: Improved Analgesia and Narcotic Use Reduction

**DOI:** 10.1002/oto2.17

**Published:** 2023-03-14

**Authors:** Carlos X. Castellanos, Marcus Paoletti, Ruben Ulloa, Celeste Kim, Michelle Fong, Meredith Xepoleas, Uttam Sinha, Niels Kokot, Mark S. Swanson

**Affiliations:** ^1^ Caruso Department of Otolaryngology‐Head & Neck Surgery Keck Medicine of University of Southern California Los Angeles California USA

**Keywords:** multimodal analgesia, opioid, postoperative pain, transoral robotic surgery

## Abstract

**Objective:**

To compare postoperative pain scores and opioid consumption in patients after transoral robotic surgery (TORS).

**Study Design:**

Single institution retrospective cohort study.

**Setting:**

TORS was performed at a single academic tertiary care center.

**Methods:**

This study compared traditional opioid‐based and opioid‐sparing multimodal analgesia (MMA) regimens in patients with oropharyngeal and supraglottic malignancy after TORS. Data were obtained from the electronic health records from August 2016 to December 2021. The average postoperative pain scores and total opioid consumption in morphine milligram equivalents were calculated for postoperative days (PODs) 0 to 3. The secondary objectives were to quantify and characterize opioid prescriptions upon hospital discharge.

**Results:**

A total of 114 patients were identified for this study, 58 patients in the non‐MMA cohort and 56 in the MMA cohort. Postoperative pain levels in the MMA cohort were statistically lower on POD 0 (*p* = 0.001), POD 1 (*p* = 0.001), and POD 3 (*p* = 0.004). Postoperative opioid consumption decreased significantly in the MMA cohort from 37.7 to 10.8 mg on POD 0 (*p* = 0.002), 65.9 to 19.9 mg on POD 1 (*p* < 0.001), 36.0 to 19.3 mg on POD 2 (*p* = 0.02), and 45.4 to 13.8 mg on POD 3 (*p* = 0.02). The number of patients discharged from the hospital with a prescription for narcotics was significantly lower in the MMA cohort (71.4%) compared with the non‐MMA cohort (98.3%) (*p* < 0.001).

**Conclusion:**

Implementation of our MMA pain protocol reduced pain levels and narcotic consumption in the immediate postoperative period.

While multimodal analgesia (MMA) protocols have existed for over 2 decades for pain management, they have become more widely adopted due to the opioid crisis.^1^ MMA regimens harness multiple nonopioid analgesics with varying pain palliating mechanisms that work synergistically to moderate pain. Previously published MMA regimens for head and neck (H&N) surgery document the use of acetaminophen, celecoxib, gabapentin, ketamine, and ketorolac.^2^, ^3^, ^4^ Under MMA regimens, nonopioid analgesics can be administered perioperatively to decrease opioid use while effectively reducing patient‐reported pain levels. Avoiding conventional opioid‐based protocols can limit opioid misuse and circumvent opioid‐associated medical complications in head and neck cancer (HNC) patients with poor functional status.

MMA is a central component of enhanced recovery after surgery (ERAS) pathways, which aim to optimize patient outcomes postoperatively.^5^ ERAS pathways harnessing MMA have roots in colorectal surgery but have been embraced by a growing number of surgical fields.^6^, ^7^ Recently, ERAS pathways incorporating MMA have been utilized in HNC treatment, particularly in tissue transfer procedures.^5^ For instance, Vu et al^3^ found a 21.5% reduction in opioid use in the PACU after H&N free flap reconstruction. Clark et al^4^ documented a decrease in opioid consumption in each of the first 5 postoperative days (PODs) ranging from 80% to 83%, and a reduction in postoperative pain in the first 2 PODs. Additionally, several studies piloting an H&N ERAS pathway reported a reduction in narcotics prescribed to patients at discharge.^8^, ^9^, ^10^

However, there are few studies on MMA protocols for trans‐oral robotic surgery (TORS). TORS has become a primary treatment modality for early‐stage oropharyngeal cancers.^11^ Surgical ablation of the oropharynx is traditionally associated with high opioid requirements for pain management, highlighting the potential role of MMA in addressing postoperative pain in these patients.^12^ Recently, Ganti et al^13^ published promising data on an ERAS protocol addressing perioperative pain in 19 patients after TORS. However, it is still unclear how MMA in isolation impacts outcomes in TORS, or how outcomes may vary in other settings. Thus, the purpose of this study is to investigate the impact of our institution's MMA regimen on postoperative pain scores and opioid requirements in TORS patients.

## Methods

### Study Population

The University of Southern California (USC) Institutional Review Board approved our retrospective cohort study (IRB Number: HS‐20‐00921). HNC patients undergoing planned TORS for primary SCC of the oropharynx and supraglottic larynx were consecutively enrolled from June 2019 to December 2021 in the MMA cohort. A retrospective control cohort of patients from August 2016 to May 2018 who received identical procedures but were not treated with an MMA protocol were enrolled consecutively for comparison. These procedures were performed at a single academic tertiary care center where patients were operated on by 1 of 3 microvascular trained otolaryngologists at the Keck Medical Center of USC.

All subjects were over the age of 18 and underwent first‐time TORS for oropharyngeal and supraglottic laryngeal malignancy. Pregnancy was an exclusionary criterion. Patients undergoing repeat TORS, and those receiving TORS for nonmalignant etiologies were excluded from the study. Patients with a history of minor H&N surgery, H&N radiation, chemotherapy, and prior exposure to opiates were included.

### H&N Postoperative MMA Management

**Figure 1:**
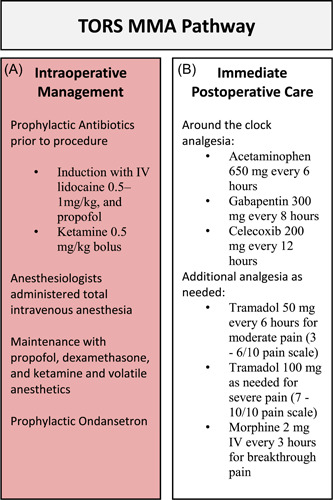
Multimodal anesthesia protocol. (A) Intraoperative management. (B) Immediate postoperative care. IV, intravenous; MMA, multimodal analgesia; TORS, transoral robotic surgery.

The H&N ERAS protocol was created by collaborative efforts between the departments of otolaryngology and anesthesia at the Keck Medical Center of USC. The H&N protocol integrates the core principles outlined by Dort et al at the ERAS society in 2017^5^ and was fully unveiled in August 2018. An in‐depth overview of the Keck Medical Center's H&N ERAS protocol can be found in previously published work from our institution.^4^ To date, only the MMA portion of the ERAS pathway has been extended to TORS patients in June 2019 and is the primary focus of this study. The medications that comprise the MMA pain regimen, outlined in Figure 1, are administered around the clock in the immediate postoperative period and consist of acetaminophen, gabapentin, and celecoxib. Tramadol and morphine are reserved for moderate and severe breakthrough pain, respectively. In the immediate postoperative period, patients are routinely admitted to the intensive care unit (ICU) overnight, where they receive one‐to‐one care by an intensive‐care nurse and around‐the‐clock analgesia. Extended postoperative care occurs in the otolaryngology‐specific step‐down unit, where patients undergo swallow assessments and work with dieticians to advance their diet. The MMA pain protocol is a standardized, premade order set attributed to every TORS patient in the MMA cohort. As the complete ERAS protocol is yet to be unveiled for TORS patients, discharge medications were largely based on surgeon preference and often mirrored an individual patient's in‐hospital pain regimen.

### Data Collection

Preoperative data collected from our institution's electronic medical records (EMR) for secondary analysis included age, sex, alcohol and tobacco use, Charlson Comorbidity Index, prior history of H&N radiation, chemotherapy, and opiate use. Operative data collected included the primary site of malignancy. Postoperative data collected included surgical complications, final pathologic T‐ and N‐stage, patient‐reported numeric pain scores, strength and dose of opiate medication prescribed at discharge, admissions within 30 days, and emergency department admissions. Pain scores were reported by patients using an 11‐point pain scale ranging from 0 to 11 (0 = no pain, 1‐3 = mild, 4‐6 = moderate, 7‐9 = severe, 10 = worst imaginable pain) and recorded in the EMR by nursing staff. Patient controlled analgesia was not included in our analysis as these values are not uniformly recorded in our institution's EMR.

Opioid consumption was converted to morphine milligram equivalents (MME), a standardized reporting scheme utilized in previously published work.^14^ Discharge narcotic prescriptions were also recorded, with a distinction between strong and weak opioids for the purposes of secondary analysis. The categorization of strong (hydrocodone, oxycodone, methadone, hydromorphone, oxymorphone, morphine) versus weak (tramadol, codeine, meperidine) narcotics reflected the guidelines established by the World Health Organization (WHO) analgesic ladder.^15^

### Outcomes

The primary outcomes of this analysis were pain scores and opioid consumption documented within the first 72 hours postoperatively. Secondary outcomes were opioid prescribing patterns upon hospital discharge, hospital length of stay (LOS), and ICU LOS.

### Statistical Analysis

Statistical analysis was performed using SPSS Statistics software (IBM Corporation, Version 24). Descriptive statistics, including means, standard deviations, medians, and ranges, were used when appropriate. Pearson's *χ* ^2^ and Fisher's exact test were used to analyze categorical variables and associations between treatment groups. Student's *t*‐tests were utilized to analyze continuous variables. Statistical significance was established as *p* < 0.05.

## Results

A total of 114 patients were identified for this study, 58 patients in the non‐MMA cohort and 56 in the MMA cohort. The combined study population was 97 males (85.1%), 78 Caucasian (69%), and 101 (91.8%) non‐Hispanic or Latino patients. There were no significant differences in race (*p* = 0.6), preoperative opioid use (*p* = 0.9), history of H&N irradiation (*p* = 1.0), T‐stages 1 through 4 (*p* = 0.8, 0.8, 0.9, 0.6), primary site of malignancy (palatine tonsil: *p* = 0.2, the base of tongue: *p* = 0.2, larynx: *p* = 1.0, and other oropharyngeal: *p* = 0.9), human papilloma virus status (*p* = 1.0) and surgical complications. Demographic and hospital data are shown in Table 1.

**Table 1:** Preoperative Cohort Demographics and Clinical Characteristics

Characteristic	Total, n = 114, n (%)	Non‐MMA, n = 58, n (%)	MMA, n = 56, n (%)	*p* value
Age, y				
Mean value		61.7	64.6	0.138
Sex				0.733
Female	17 (14.9)	8 (13.8)	9 (16.1)	
Male	97 (85.1)	50 (86.2)	47 (83.9)	
Race				0.626
Asian	4 (3.5)	3 (5.3)	1 (1.8)	
Caucasian	78 (69.0)	40 (70.2)	38 (67.9)	
Black	6 (5.3)	2 (3.5)	4 (7.1)	
Unknown	25 (22.1)	12 (21.1)	13 (23.2)	
Ethnicity				0.033
Hispanic or Latino	9 (8.2)	8 (14.0)	1 (1.9)	
Non‐Hispanic or Latino	101 (91.8)	49 (86.0)	52 (98.1)	
Smoker status				0.133
Current	10 (8.8)	7 (12.1)	3 (5.4)	
Former	47 (41.2)	27 (46.6)	20 (35.7)	
Never	57 (50.0)	24 (41.4)	33 (58.9)	
Alcohol status				0.183
Current	69 (60.5)	34 (58.6)	35 (62.5)	
Former	12 (10.5)	8 (13.8)	4 (7.1)	
Never	30 (26.3)	13 (22.4)	17 (30.4)	
Not reported	3 (2.6)	3 (5.2)	0 (0.0)	
Preoperative opioid use				0.974
Yes	12 (10.6)	6 (10.5)	6 (10.7)	
No	101 (89.4)	51 (89.5)	50 (89.3)	
Previous chemotherapy				0.679
Yes	6 (5.3)	4 (6.9)	2 (3.5)	
No	108 (94.87)	54 (93.1)	54 (96.4)	
Previous irradiation				1.00
Yes	7 (6.1)	4 (6.9)	3 (5.3)	
No	108 (93.9)	54 (93.1)	54 (94.7)	
pT‐stage				
1	42 (40.0)	21 (38.9)	21 (41.2)	0.811
2	44 (41.9)	22 (40.7)	22 (43.1)	0.804
3	15 (14.3)	8 (14.8)	87 (13.7)	0.873
4	4 (3.8)	3 (5.6)	1 (2.0)	0.618
pN‐stage				
0	20 (18.3)	7 (12.5)	13 (24.5)	0.105
1	39 (35.8)	15 (26.8)	24 (45.3)	0.044
2	29 (26.6)	20 (35.7)	9 (17.0)	**0.027**
3	5 (4.6)	5 (8.9)	0 (0.0)	0.057
Unknown	16 (14.7)	9 (16.1)	7 (13.2)	0.673
Primary site				
Palatine tonsil	41 (36.3)	18 (31.0)	23 (41.8)	0.233
Base of tongue	54 (47.8)	31 (53.4)	23 (41.8)	0.216
Larynx	6 (5.3)	3 (5.2)	3 (5.5)	1.00
Other oropharyngeal	12 (10.6)	6 (10.3)	6 (10.9)	0.922
HPV status				1.00
Positive	90 (93.8)	44 (93.6)	46 (93.9)	
Negative	6 (6.3)	3 (6.4)	4 (6.1)	
Complication				
Wound dehiscence	0 (0.0)	0 (0.0)	0 (0.0)	–
Fistula—saliva leakage	0 (0.0)	0 (0.0)	0 (1.0)	–
Wound infection	0 (0.0)	0 (0.0)	0 (0.0)	
Hematoma	2 (1.8)	0 (0.0)	2 (3.6)	0.239
Death	1 (0.9)	0 (0.0)	1 (1.8)	0.491
ED readmission				1.00
Yes	2 (1.8)	1 (1.7)	1 (1.8)	
No	111 (98.2)	57 (98.3)	54 (98.2)	

Statistically significant *p* values are bolded. Analyzed but not included in the table due to nonsignificance include discharge to destination other than home and modified radical neck dissection

Abbreviations: ED, emergency department; HPV, human papilloma virus; MMA, multimodal anesthesia.

### Pain Levels and Opioid Requirements

Average postoperative pain levels in the MMA cohort were statistically lower on POD 0 (*p* = 0.001), POD 1 (*p* < 0.001), and POD 3 (*p* < 0.001). The overall average postoperative pain levels (mean; SD) were significantly lower (*p* < 0.001) in the MMA group (2.6; 1.7) compared with the non‐MMA group (4.1; 1.4) with a large effect size (*d* = 1.02). Postoperative opioid consumption (MME) decreased significantly in the MMA cohort from an average of 37.7 to 10.8 mg on POD 0 (*p* = 0.002), 65.9 to 19.9 mg on POD 1 (*p* = 0.001), 36.0 to 19.3 mg on POD 2 (*p* = 0.02), and 45.4 to 13.8 mg on POD 3 (*p* = 0.02). Average MMEs consumed postoperatively (mean; SD) were significantly lower (*p* < 0.001) in the MMA group (15.5; 18.7) compared with the non‐MMA group (43.5; 47.3) with a large effect size (*d* = 0.81). There was no significant reduction in ICU (*p* = 0.3) and hospital (*p* = 0.1) length of stay between each cohort. The primary and secondary objectives of this study are outlined in Table 2.

**Table 2:** Primary and Secondary Outcomes

Characteristic	Non‐MMA	MMA	95% CI of the difference	*p* value
ICU LOS	0.6	1.7	[−3.0, 0.8]	0.260
Hospital LOS	3.4	5.1	[−3.5, 0.2]	0.08
Pain				
POD 0 average	4.5	3.1	[0.6, 2.3]	**0.001**
POD 0 peak	7.7	5.5	[1.0, 3.5]	**0.001**
POD 1 average	4	2.2	[1.1, 2.4]	**<0.001**
POD 1 peak	7.3	5.2	[1.1, 3.1]	**<0.001**
POD 2 average	3.7	2.8	[−0.5, 2.4]	0.19
POD 2 peak	6.7	5.4	[0.3, 2.3]	**0.012**
POD 3 average	3.8	2.0	[1.0, 2.7]	**<0.001**
POD 3 peak	6.5	4.5	[0.7, 3.5]	**0.004**
Total average	4.1	2.6	[0.9, 2.1]	**<0.001**
MME consumed				
POD 0	37	10.8	[10.3, 42.1]	**0.002**
POD 1	64.8	20	[20.3, 69.2]	**0.001**
POD 2	36	19.3	[1.9, 31.5]	**0.027**
POD 3	45.4	13.8	[5.2, 58.8]	**0.021**
Total average	43.5	15.5	[14.6, 41.5]	**<0.001**

Statistically significant *p* values are bolded.

Abbreviations: CI, confidence interval; LOS, length of stay; MMA, multimodal anesthesia; MME, morphine milligram equivalents; POD, postoperative day.

### Opioid Prescriptions at Hospital Discharge

The number of patients discharged from the hospital with a prescription for narcotics was significantly lower (*p* < 0.001) in the MMA cohort (71.4%) compared with the non‐MMA cohort (98.3%) (Table 3). Significantly fewer (*p* < 0.001) patients (number; percentage) in the MMA cohort (16; 40%) were discharged with strong opiates compared with the non‐MMA cohort (50; 87.7%). Patients in the MMA (22; 39.3%) cohort were discharged with significantly more prescriptions for Tramadol (*p* = 0.004) than the non‐MMA cohort (9; 15.5%). The MMA cohort (14; 25%) received significantly fewer hydrocodone prescriptions (*p* < 0.001) at discharge than the non‐MMA cohort (45; 77.6%). The total average MMEs prescribed at hospital discharge for the MMA (203.6) was less than the non‐MMA (252.1), but not significantly reduced (*p* = 0.4) (Table 4).

**Table 3:** Opioid Prescription Characteristics at Hospital Discharge

Characteristic	Total, n (%)	Non‐MMA, n (%)	MMA, n (%)	*p* value
Discharged with opioids				**<0.001**
Yes	97 (85.1)	57 (98.3)	40 (71.4)	
No	17 (14.8)	1 (1.7)	16 (28.1)	
Opioid strength^a^				**<0.001**
Strong	66 (68.0)	50 (87.7)	16 (40.0)	
Weak	31 (32.0)	7 (12.3)	24 (60.0)	
Medication^b^				
Tramadol	31 (27.2)	9 (15.5)	22 (39.3)	**0.004**
Hydrocodone	59 (51.8)	45 (77.6)	14 (25.0)	**<0.001**
Hydromorphone	1 (0.9)	1 (1.7)	0 (0.0)	1.000
Oxycodone	10 (8.8)	6 (10.3)	4 (7.1)	0.743
Oxymorphone	0 (0.0)	0 (0.0)	0 (0.0)	

Statistically significant *p* values are bolded.

Abbreviation: MMA, multimodal anesthesia.

^a^ Strong opioids: Hydrocodone, oxycodone, methadone, hydromorphone, oxymorphone, morphine, and buprenorphine. Weak opioids: Tramadol, codeine, and meperidine.

^b^ Sum greater than 100% as some patients received multiple medications.

**Table 4:** Opiates Prescription Characteristics by Medication Type

	Average prescribed MME at discharge		
Discharge opioid	Non‐MMA	MMA	*p* value
Tramadol, tablets	146.9	135.9	0.784
Hydrocodone, mL	226.0	207.5	0.550
Hydrocodone, tablets	263.7	146.3	0.092
Hydrocodone, total	242.3	172.5	0.061
Oxycodone, mL^a^	720.0	0	
Oxycodone, tablets	328.1	783.8	0.528
Oxycodone, total	406.5	783.8	0.599
All opioids	252.1	203.6	0.421

Abbreviations: MMA, multimodal anesthesia; MME, morphine milligram equivalents.

^a^ *p* value not calculated because 1 group is empty.

## Discussion

Our study revealed a statistically significant reduction in the immediate postoperative pain levels and opioid requirements for patients with oropharyngeal and supraglottic SCC surgically treated with TORS. Average pain levels on POD 0, 1, and 3 were significantly lower in the MMA cohort compared with the non‐MMA cohort. Total pain levels averaged throughout the hospital admission for the MMA cohort were significantly reduced compared with the non‐MMA cohort with a large effect size (*d* = 1.02). Similarly, postoperative opioid consumption (MME) decreased significantly in the MMA cohort on POD 0, 1, 2, and 3. Average MMEs consumed postoperatively were significantly lower in the MMA group compared with the non‐MMA group with a large effect size (*d* = 0.81).

Narcotics have historically served as the cornerstone of pain protocols and can effectually moderate pain, but carry potentially deleterious side effects, including respiratory depression, nausea and vomiting, impaired mobilization, and the possibility of chronic dependence. Up to 80% of HNC patients undergoing surgical treatment with adjuvant therapy experience pain requiring prescription opioids.^16^ Paradoxically, patient satisfaction with narcotic‐based pain regimens is low, with 70% to 85% of patients reporting ineffective pain control.^17^ Moreover, the surgical approaches to address HNC range from minimally invasive TORS to extensive ablation with tissue transfer reconstruction. Interestingly, the scale of surgical resection may not predictably correlate with chronic opiate consumption following treatment.^18^ Perhaps a more useful predictor of postoperative pain relies on the anatomic subsite. Patient‐reported pain levels are the highest, and opioid prescribing patterns among otolaryngologists are predominant in patients undergoing surgical ablation in the oropharynx.^12^, ^19^, ^20^ Consequently, the prevalence of persistent postoperative opioid use for these patients can be high. A study by McDermott et al surveying Medicare patients treated for oral cavity and oropharyngeal cancer between 2008 and 2011 found that 15.4% had continued opioid use at 3 months and 7% at 6 months after discharge.^21^ Recently, Hinther et al^22^ reported chronic opioid use in 52% of opioid‐naïve patients and 82% of preoperative opioid users following HNC treatment.

MMA is an important paradigm shift in the approach to limiting chronic opiate misuse and preventing opioid‐related deaths.^2^, ^23^ MMA is a synergistic drug regimen aimed at triggering different components of an anesthetic state: amnesia, akinesia, and anti‐nociception. Commonly cited medication in previous work includes gabapentin, nonsteroidal anti‐inflammatory drugs, *N*‐methyl‐d‐aspartate class of glutamate receptor antagonists, lidocaine, and regional anesthetic blocks.^24^ Studies on the use of MMA to reduce opioids show promising but mixed results. A systematic review by Go et al^25^ found that of 10 studies that used MMA for H&N free flap patients, 8 had significantly less opioid use, but 2 studies did not. Moreover, the studies explored by Go et al's revealed marked heterogeneity among MMA pathways, multifariously including gabapentin (73%), nonsteroidal anti‐inflammatory drugs (45%), acetaminophen (44%), corticosteroids (25%), and ketamine (7%), as well as lower extremity nerve blocks (3%).^25^ Nevertheless, there remains a promising role for MMA in addressing pain for HNC patients. Thus, there is a continued need to investigate the relationship between MMA and its effects on opioid use across specific sites of malignancy and various surgical approaches.

TORS has become the surgical treatment of choice for early‐stage oropharyngeal cancers due to its minimally invasive nature, high accuracy regarding the staging of primary disease, and excellent swallowing outcomes.^11^ Contributors to chronic opioid use are multifactorial, but studies focusing on HNC acknowledge a few key predisposing factors which involve preoperative opiate use, history of tobacco use, race, radiation therapy, and advanced pathologic t‐stage^10^, ^18^, ^26^, ^27^ Many of these factors apply to patients in our research cohorts treated with TORS. As previously mentioned, surgery of the oropharynx is associated with high levels of postoperative pain and resultant opioid prescriptions. Furthermore, oropharyngeal cancer shares 2 independent risk factors with long‐term opioid use—cigarette and alcohol use. In the patient population examined in this study, 50.4% of patients were either current or former smokers, and 70.4% had current or previous use of alcohol. Therefore, TORS patients often require opioids and tend to have an elevated risk of developing long‐term opioid use.

While postanesthesia recovery time, ICU, and hospital LOS are commonly explored in H&N MMA and ERAS literature, the impact on these variables has been variable.^2^, ^8^, ^13^, ^28^ In this study, there was a reduction in ICU and hospital length of stay between each cohort, albeit a nonsignificant one despite the effectiveness of MMA in reducing pain and opioid consumption. In contrast to comprehensive care pathways like ERAS, MMA is a singular component aimed at addressing pain. Optimizing a patient's care pathway throughout their hospital stay may require a more holistic and concerted approach to ERAS protocols. The routine admission to the ICU following TORS in our department and administrative restraints of hospital discharge planning may have also contributed to a lack of significant reduction in these secondary endpoints. Importantly, there was no significant difference in postoperative complication between each cohort, including the incidence of postoperative bleeding. These data support the safety of celecoxib use in MMA protocols demonstrated in the breast and plastic surgery literature.^29^, ^30^

Upon hospital discharge, there was a significant decline in patients discharged with any opioid medication in the MMA cohort (*p* < 0.001). This trend continued when the prescribed opioids were dichotomized as strong or weak, in accordance with WHO and DEA regulations, showing MMA patients receiving significantly fewer strong opioids (*p* < 0.001). Specifically accounting for this result was a department‐wide shift in practice to discharge MMA patients with tramadol (non‐MMA: 15.5% vs MMA: 39.3%; *p* = 0.004) in the place of previously opted for hydrocodone (non‐MMA: 77.6% vs MMA: 25%; *p* < 0.001). This prescription pattern in the non‐MMA is not unique to our institution,^12^, ^19^ but served as a potential practice to improve upon. When comparing average MMEs prescribed between cohorts, we found a nonsignificant (*p* = 0.4) reduction in the MMA cohort (203.6 mg) compared with the non‐MMA cohort (252.1 mg). Reflecting on this data is essential to the manner our department addresses postoperative pain for TORS patients. If lower postoperative pain levels can be achieved with restrained use of narcotics, then perhaps patients do not require routine opioid prescriptions at discharge and should be evaluated more carefully.

A robust pain protocol is intimately linked with postoperative surgical outcomes. Ineffectually addressing postoperative pain may contribute to prolonged immobility, poor functional outcomes, and secondarily to wound infection, modest wound healing, extended hospital stays, and diminished quality of life.^31^ Furthermore, suboptimal pain pathways can theoretically lead to reduced adherence to adjuvant oncologic therapies. Existing literature highlights inadequate pain relief in HNC patients whose regimen relied on narcotics.^26^, ^27^

Our study adds to previously published work supporting the benefit of MMA protocols for robotic‐assisted surgery.^32^, ^33^, ^34^ Our study builds on work by Ganti et al by exploring the impact of MMA on HNC patients treated with TORS on pain and narcotic requirements in each isolated POD among a larger patient cohort. Furthermore, work by Abt et al revealed total MME usage from the start of treatment, TORS with or without adjuvant treatment, averaged 1395.7 mg with 76.4% of patients receiving 3 opioid prescriptions or less.^31^ Combined in‐hospital and prescription MME totals consumed for the MMA and non‐MMA cohorts in our study were 219.3 and 295.6 mg, respectively. These values highlight the post‐hospital‐discharge period as a critical window during which a majority of MMEs are consumed in a patient's treatment journey and a key time frame to address in future work seeking to limit opioid exposure in this patient population. Special attention must be given when quantifying opioid consumption as the equivalency factors and time frame referenced depend on the reporting scheme.^14^ Our group advocate for MMEs as they consider drug quantity, time supply, and published equivalency factors assigned to opioid medications that ultimately facilitate comparison of opioid data in the scientific literature.^35^, ^36^

This study has limitations warranting further discussion. The retrospective cohort design of this study may introduce a level of bias in so far as at the time of treatment, the attending physicians and residents were aware of the pain regimen each patient was assigned to. This knowledge could have raised the threshold at which providers were likely to order narcotics to address postoperative pain in the hospital and at discharge. We report on patient cohorts with primary SCC in various anatomic subsites of the oropharynx and larynx. The heterogeneity in disease sites may contribute to differences in the subjective experience of postoperative pain. However, the sites of primary malignancy did not vary between each cohort. When evaluating the impact of MMA on pain and opioid requirements among HNC patients, the variety in surgical procedures and heterogeneity in MMA protocols referenced in the literature contribute to the difficulty in comparing data in the relevant literature.

## Conclusion

The outlined MMA regimen is effective for pain management and opioid use reduction in the immediate postoperative period for patients treated with TORS for primary SCC of the oropharynx and supraglottic larynx at our institution. Additionally, the patient cohort treated with an MMA protocol was prescribed fewer strong‐classification opioids and overall opioids with no differences in postoperative complications, ICU, and hospital length of stay. The addition of MMA pain pathways in H&N surgery is a relatively new, yet a promising approach that will benefit from long‐term surveillance and constant reflection on prescribing practices and patient outcomes.

## Author Contributions

**Carlos X. Castellanos**, drafting the work, agreement to be accountable for all aspects of the work in ensuring that questions related to the accuracy or integrity of any part of the work are appropriately investigated and resolved, acquisition of data, conception of the work, data analysis, revision of the work critically for important intellectual content; **Marcus Paoletti**, drafting the work, agreement to be accountable for all aspects of the work in ensuring that questions related to the accuracy or integrity of any part of the work are appropriately investigated and resolved, acquisition of data, data analysis; **Ruben Ulloa**, agreement to be accountable for all aspects of the work in ensuring that questions related to the accuracy or integrity of any part of the work are appropriately investigated and resolved, data analysis; **Celeste Kim**, agreement to be accountable for all aspects of the work in ensuring that questions related to the accuracy or integrity of any part of the work are appropriately investigated and resolved, acquisition of data, conception of the work, data analysis; **Michelle Fong**, agreement to be accountable for all aspects of the work in ensuring that questions related to the accuracy or integrity of any part of the work are appropriately investigated and resolved, acquisition of data; **Meredith Xepoleas**, agreement to be accountable for all aspects of the work in ensuring that questions related to the accuracy or integrity of any part of the work are appropriately investigated and resolved, acquisition of data; **Uttam Sinha**, agreement to be accountable for all aspects of the work in ensuring that questions related to the accuracy or integrity of any part of the work are appropriately investigated and resolved, revision of the work critically for important intellectual content, final approval of the version to be published; **Niels Kokot**, conception of the work, agreement to be accountable for all aspects of the work in ensuring that questions related to the accuracy or integrity of any part of the work are appropriately investigated and resolved, revision of the work critically for important intellectual content, final approval of the version to be published; **Mark S. Swanson**, conception of the work, acquisition of data, agreement to be accountable for all aspects of the work in ensuring that questions related to the accuracy or integrity of any part of the work are appropriately investigated and resolved, revision of the work critically for important intellectual content, final approval of the version to be published.

## Disclosures

### Competing interests

None.

### Sponsorships

None.

### Funding source

None.
